# Outcomes of an In Vitro Fertilization Pregnancy With COVID-19 and the Perinatal Outcome in Riyadh, Saudi Arabia

**DOI:** 10.7759/cureus.12296

**Published:** 2020-12-26

**Authors:** Abdullah H Alhamoud, Faeza Matary, Shaheera Bukhari, Mashael Kelantan, Mohammed Bajahzer

**Affiliations:** 1 Medicine, College of Medicine, King Saud Bin Abdulaziz University for Health Sciences College of Medicine, Riyadh, SAU; 2 Laboratory Medicine, Al-Imam Abdulrahman Al-Faisal Hospital, Ministry of Health, Riyadh, SAU; 3 Obstetrics and Gynaecology, Al-Imam Abdulrahman Al-Faisal Hospital, Ministry of Health, Riyadh, SAU; 4 Medicine, School of Population and Global Health, The University of Melbourne, Melbourne, AUS; 5 Public Health, Ministry of Education, Riyadh, SAU; 6 Clinical Nutrition, Applied Medical Sciences, Jazan University, Jazan, SAU

**Keywords:** covid-19, in vitro fertilization ivf, pregnancy, vertical transmission

## Abstract

Coronavirus disease 2019 (COVID-19) caused by the novel severe respiratory syndrome coronavirus 2 (SARS-CoV-2) is a pandemic and potentially fatal disease. COVID-19 cases are on the rise globally; this also includes risk groups such as pregnant women and neonates. Herein, we report the first COVID-19 cesarean delivery case of an in vitro fertilization (IVF) pregnancy in a Saudi woman. A postdate pregnant healthy woman tested positive with COVID-19 on her 38 weeks + five days. On her 40 weeks + five days, the woman had dilation without contractions; thereby, cesarean delivery was decided. The delivery was successful, with no complications in the mother and neonate. The preferable outcomes of this case could be attributable to some factors: multidisciplinary medical management, the mother’s young age, and COVID-19 infection during the late trimester.

## Introduction

On March 11, 2020, the World Health Organization (WHO) declared the current pandemic of COVID-19 caused by SARS-Cov-2. Thus, rapid medical and scientific efforts are being made to obtain further knowledge of the disease. However, limited data are available on the consequences of the disease on some risk groups such as pregnant women and neonates. COVID-19 comprises acute respiratory symptomatology that can range from mild to critical, and, in some cases, could be fatal. To date, a small case series from China [1] yields most of the data available on COVID-19 interaction with pregnancy. The pregnant women were reported with mild symptoms of COVID-19, whereas no evidence of mother to fetus transmission [2]. Noteworthy, the risk is higher for pregnant women to develop severe viral pneumonia compared to the general public. Indeed, the mortality rate of SARS-CoV infection in this particular population was reported at around 10% [3]. Herein, we report the first case cesarean delivery of in-vitro fertilization (IVF) pregnancy with COVID-19 in Riyadh, KSA. The case was treated and followed at Al-Imam Abdurrahman Al-Faisal hospital since it was diagnosed with COVID-19.

## Case presentation

We report here a case of a 27-year-old female in her second pregnancy with no previous history of chronic illness. She has had a laparotomy due to right ovarian torsion. Additionally, she had a miscarriage in the first trimester of her first pregnancy due to unknown causes and without complications. The IVF technique was used to induce her second pregnancy (the current) due to secondary infertility and the male factor. She was admitted at 38 weeks + five days gestation due to an interaction with a confirmed case of COVID-19. Two consecutive COVID-19 positive nasopharyngeal swabs confirmed her COVID-19 infection. The reverse transcription-polymerase chain reaction (RT-PCR) was the method of analysis for COVID-19. All of her basic blood tests, electrocardiogram, and chest X-ray with an abdominal shield were unremarkable. The patient chest examination showed equal bilateral air entry, no crepitation, and no wheezing. The patient cardiovascular examination S1+ S2 showed no added sound. All tests were routinely repeated, as needed, which showed a good prognosis without needing specific treatment for the infection. The patient was clinically and vitally stable; thus, she received no treatment for COVID-19. Two consecutive COVID-19 negative nasopharyngeal swabs confirmed her COVID-19 recovered. At 41 weeks + five days gestation, the patient was transferred to the delivery room after showing signs of beginning labor. A cesarean section (CS) was later decided due to failure to progress and pathological cardiotocography (CTG), as shown in the CTG. All precautions of personal protective equipment (PPE) were taken to make all health providers are safe. The CS was successfully carried under spinal anesthesia without complications. A postdate baby boy weighing 3.9 kgs was delivered at 41 weeks + five days with no complications. His APGAR (Appearance, Pulse, Grimace, Activity, and Respiration) scores were 7 and 9 at one and five minutes, respectively. The baby was then transferred to the Special Intensive Care Unit. The mother was overall well and, thus, was discharged. Two consecutive COVID-19 negative nasopharyngeal swabs confirmed the baby boy to be clear of COVID-19.

## Discussion

Here, we reported the clinical course of COVID-19 infection in an IVF pregnant woman who was infected during the last trimester. This was her second delivery and the first cesarean without complications for the mother and neonate.

The IVF technique is the method of choice offered when it is difficult to conceive naturally. Multiple pregnancies, miscarriages, and obstetrical complications are risks that might affect IVF pregnancies, whereas, neonatal prematurity and morbidity are risks that associate more with IVF neonates [4]. There is an additional risk of ovarian hyperstimulation syndrome if human chorionic gonadotropin is used to induce oocyte maturation [5]. Additionally, wounds, bleeding, and infections are possible risks of transvaginal ovum retrieval and laparoscopy. The ultrasound aspiration might also occur, especially in the bowel and the bladder. Other common complications might occur such as breathing difficulty, chest infection, and allergic reactions to drugs [6].

The whole population is exposed to the risk of the COVID-19 pandemic, including risk groups such as pregnant women and neonates. To this end, COVID-19 is observed to be less severe in pregnant women than SARS-CoV and Middle East respiratory syndrome coronavirus (MERS-CoV) infections [2]. Nonetheless, the vertical transmission of such diseases is a possible risk if pregnant women are infected. The available data of different body samples from COVID-19 retrospective studies were negative for SARS-CoV-2 [7-9]. Collectively, the available COVID-19 data suggest that intrauterine transmission to the fetus is unlikely [10-11].

To the best of our knowledge, this is the first case report of an IVF pregnancy with COVID-19 infection and the neonatal outcome in Riyadh, KSA. Herein, the clinical indications of both the mother and the infant proved them healthy. The neonate's negative COVID-19 tests suggest no intrauterine transmission. However, excluding the risk of SARS-CoV-2 vertical transmission cannot be generalized and excluded.

## Conclusions

In conclusion, there is no evidence to suggest a higher risk of COVID-19 vertical transmission in IVF pregnancies. Optimally, it is crucial to follow interdisciplinary medical management to counter possible complications. Moreover, the limited data on COVID-19 and its effect on pregnancies and neonates warrant further research.
